# The role of pyrope garnet in water transport into the topmost lower mantle

**DOI:** 10.1093/nsr/nwaf133

**Published:** 2025-04-04

**Authors:** Luyao Chen, Xinyu Zhao, Chaowen Xu, Xin Li, Xinran Zhang, Bingtao Feng, Kuo Hu, Bingbing Liu, Zhaodong Liu, Wenliang Xu

**Affiliations:** State Key Laboratory of High Pressure and Superhard Materials, Synergetic Extreme Condition User Facility, Jilin University, Changchun 130012, China; State Key Laboratory of High Pressure and Superhard Materials, Synergetic Extreme Condition User Facility, Jilin University, Changchun 130012, China; Institute of Earthquake Forecasting, China Earthquake Administration, Beijing 100036, China; State Key Laboratory of High Pressure and Superhard Materials, Synergetic Extreme Condition User Facility, Jilin University, Changchun 130012, China; College of Chemistry, Jilin University, Changchun 130012, China; State Key Laboratory of High Pressure and Superhard Materials, Synergetic Extreme Condition User Facility, Jilin University, Changchun 130012, China; State Key Laboratory of High Pressure and Superhard Materials, Synergetic Extreme Condition User Facility, Jilin University, Changchun 130012, China; State Key Laboratory of High Pressure and Superhard Materials, Synergetic Extreme Condition User Facility, Jilin University, Changchun 130012, China; State Key Laboratory of High Pressure and Superhard Materials, Synergetic Extreme Condition User Facility, Jilin University, Changchun 130012, China; College of Earth Sciences, Jilin University, Changchun 130061, China; College of Earth Sciences, Jilin University, Changchun 130061, China

**Keywords:** water transport, pyrope garnet, oceanic slabs, topmost lower mantle, high pressure

## Abstract

The role of oceanic slabs in water transport to the interior of Earth depends on the water-storage capacity of their major minerals. Pyrope garnet is the dominant mineral in basaltic slabs from the crust to the topmost lower mantle, but its water-storage capacity remains controversial owing to the difficulty in growing high-quality single crystals for precise water-content analysis. Here, we systematically investigate the water solubility in pure single crystals of synthetic pyrope garnet at pressures of 5–25 GPa and temperatures of 1100–1900 K by using large-volume presses and Fourier transform infrared spectroscopy. Our results indicate that pyrope garnet can contain up to 2000 wt. ppm H_2_O with a strong dependence on pressure and temperature in the transition zone and topmost lower mantle. Therefore, pyrope garnet may serve as a vital water carrier and reservoir in the deep mantle, offering new insights into water cycling up to the topmost lower mantle.

## Teaser

Pyrope garnet, a key mineral widespread in the Earth's interior, can retain significant amounts of water, up to 2000 parts per million, from the transition zone to the topmost lower mantle. Hydrated pyrope garnet plays a crucial role in water transport and storage within subducting slabs, in which case it may affect the physical and chemical properties of the deep mantle.

## INTRODUCTION

Water plays an important role in the evolution and dynamics of Earth [1,2] and it can be stored and transported into the interior of Earth by subducting slabs from the lithosphere. Even trace amounts of water incorporation could significantly affect the chemical and physical properties of minerals and thereby the deep mantle [3,4]. The capacity of minerals within subducting slabs to store water under high-pressure and high-temperature (HPHT) conditions of the mantle is crucial for these dynamic processes. Silicate garnet is a predominant mineral in basaltic slabs, constituting ∼70 vol% from the upper mantle to the topmost lower mantle. It also takes up ∼20 vol% in the shallow upper mantle and 40 vol% in the mantle transition zone for peridotite and eclogitic mantle models [5–7]. Although garnets typically exist as complex solid solutions, pyrope (Mg_3_Al_2_Si_3_O_12_) is one of the most significant endmember garnets. Both garnet lherzolite mantle and natural garnets from eclogite xenoliths are predominantly composed of pyrope garnet [8–10]. Pyrope can form an extensive garnet solid solution by dissolving pyroxene into its structure with increasing depth from the upper mantle to the transition zone in basaltic slabs and peridotite mantle. Furthermore, pyrope garnet may promote the slab stagnation at depths of 660–1000 km and provide an additional explanation for the seismic observations in these regions [11,12]. Given the significance of pyrope garnet in the interior of Earth, the determination of its water-storage and transport capacities is vital for understanding the water cycle there.

Natural pyrope or pyrope-rich garnets have been found to contain 2–2200 wt. ppm H_2_O by using infrared spectroscopy (IR) [13–17]. These scarce natural garnets primarily originate from the crust and shallow upper mantle, although they can survive to depths corresponding to the topmost lower mantle. Previous studies have investigated the water solubility in pyrope garnet that is synthesized under HPHT conditions corresponding to the upper mantle, but their results are still debated. HPHT annealing experiments on natural pyrope garnet suggest that its water content increases from 58 to 199 wt. ppm H_2_O with increasing pressure from ambient pressure to 10 GPa at a constant temperature of 1273 K [10]. In contrast, synthetic pyrope garnet at pressures of 2–5 GPa and temperatures of 1073–1473 K contains ∼200–700 wt. ppm H_2_O [18,19]. Withers *et al.* [20] found that the water solubility in pyrope garnet at a constant temperature of 1273 K first increases to 1000 wt. ppm H_2_O with pressures of ≤5 GPa and then decreases below the detection limit of IR at pressures of >7 GPa. Mookherjee and Karato [21] showed that the water solubility in synthetic pyrope-rich garnet generally increases from 400 to 1000 wt. ppm H_2_O with increasing pressure from 5 to 9 GPa at a constant temperature of 1373 K. Hence, the correlation between its water solubility versus pressure and temperature remains unclear under upper-mantle conditions. Additionally, water solubility in pyrope garnet under the mantle transition zone and topmost lower-mantle conditions is even more poorly constrained. Katayama *et al.* [22] found that pyrope-rich garnet synthesized from a basaltic composition at 20 GPa and 1673–1773 K contained ∼1000 wt. ppm H_2_O by using secondary ion mass spectrometry, while Thomas *et al.* [23] found that Fe-rich pyrope garnet synthesized at 18 GPa and 2073 K contains 214–881 wt. ppm H_2_O. Panero *et al.* [24] showed that synthetic basaltic garnet at 25 GPa and 1873 K can contain as much as 1440 wt. ppm H_2_O. Recently, Liu *et al.* [25] quantified the water solubility in majoritic garnets with a lower alumina content than that of pyrope to be 368–2606 ppm H_2_O at 1670–2270 K under a constant pressure of 20 GPa. However, these previous studies were mostly conducted under certain HPHT conditions. The water-storage capacity of pyrope-rich garnet as a function of pressure and temperature thus remains poorly quantified across the mantle transition zone to the topmost lower mantle due to the experimental challenges of growing high-quality single crystals of pyrope garnet under water-saturated environments for these deep-mantle conditions for precise analysis. Therefore, systematic studies are essential to elucidate the water-storage capacity of pyrope garnet under the relevant mantle pressure and temperature conditions during the subduction spanning from the upper mantle through the mantle transition zone to the topmost lower mantle.

In this study, we systematically investigated the water-storage capacity of pyrope garnet under water-saturated environments at pressures of 5–25 GPa and temperatures of 1100–1900 K by using a large-volume press and Fourier transform infrared spectroscopy (FTIR). We found that pyrope garnet can store a substantially high amount of water, particularly at depths that range from the mantle transition zone to the topmost lower mantle. Subsequently, we discussed the role of hydrous pyrope garnet in water transport in basaltic slabs and its implications for the physical and chemical properties of the mantle of Earth.

## RESULTS

### Run products

**Figure 1.**
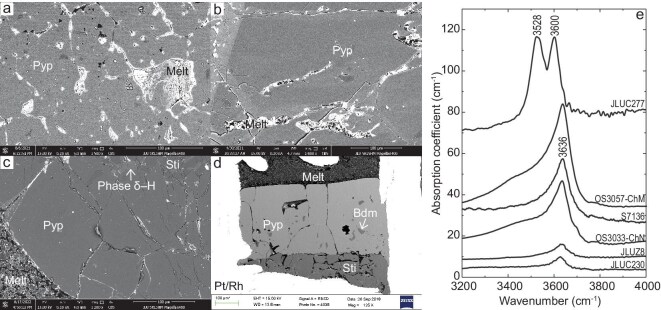
Backscattered electron images of the recovered samples. (a) JLUC224 (15 GPa and 1700 K); (b) JLUC99 (18 GPa and 1900 K); (c) JLUC312 (20 GPa and 1700 K); (d) S7136 (22 GPa and 1900 K). Pyp, pyrope; Sti, stishovite; Bdm, bridgmanite. (e) Representative FTIR spectra of pyrope in various experimental runs (JLUC230, 5 GPa and 1273 K; JLUZ8, 19 GPa and 1700 K; OS3033–ChN, 14 GPa and 1473 K; S7136, 22 GPa and 1900 K; OS3057–ChM, 25 GPa and 1873 K; JLUC277, 15 GPa and 1100 K). Spectra are offset vertically for clarity.

The experimental conditions, phase assemblage, sample thicknesses and water solubility in pyrope garnet are listed in Table 1. Single crystals of pyrope garnet are present in all recovered samples (Fig. 1a–d and Fig. S1), with grain sizes that range from 100 to 700 μm. Hydrous melts are also present in all recovered samples, which indicates that the water dissolved in the pyrope has reached its solubility limit [26], causing any excess water to exsolve as a melt or fluid phase rather than remaining in a dissolved state. At 5–12 GPa and 1273–1473 K, only pyrope coexists with hydrous melts, yet trace amounts of phase egg are observed at 7 GPa and 1273 K. At 14–24 GPa and 1100–1900 K, hydrous phases such as phase E, superhydrous phase B, phase egg, phase δ–H, phase D or stishovite coexist with pyrope in the recovered samples. At 22–25 GPa and 1873–1900 K, bridgmanite crystals coexist with pyrope garnets and hydrous melts. Element mapping and electron probe microanalysis confirm that the composition of synthetic pyrope is homogeneous (Figs S2 and S3, and Table S1). However, the aluminum depletion in synthetic pyrope relative to its ideal chemical formula is possibly caused by the preferential partitioning of Al into corundum and hydrous phases (e.g. phase D and phase δ–H) rather than pyrope under high-pressure conditions. The phases of pyrope garnets in recovered samples were identified by using Raman spectra in Fig. S4, according to previous studies [27,28].

**Table 1.** Experimental conditions, phase assemblage and water solubility in pyrope.


						C_H_2_O_ by FTIR (wt. ppm)	
Run no.	Pressure (GPa)	Temperature (K)	Duration (h)	Phase assemblage	Thickness (μm)	Bell *et al.*, 1995	Paterson, 1982

JLUC230	5	1273	24	Pyp + Melt	220	323 (48)	156 (29)
JLUC305	7	1273	24	Pyp + Phase Egg + Melt	160	486 (67)	216 (40)
JLUC268	10	1273	36	Pyp + Melt	220	1622 (671)	617 (226)
JLUC236	12	1273	40	Pyp + Melt	160	1345 (419)	500 (168)
JLUC244	12	1473	7	Pyp + Melt	200	1196 (362)	468 (83)
OS3033–ChM	14	1473	3	Pyp + Phase E + Superhydrous phase B + Melt	176	1663 (362)	773 (218)
OS3033–ChN	14	1473	3	Pyp + Phase E + Melt	144	1500 (249)	741 (136)
JLUC277	15	1100	12	Pyp + Sti + Phase Egg + Crn + Mgs + Melt	80	2046 (765)	797 (313)
JLUC364	15	1600*	12	Pyp + Crn + Superhydrous phase B + Melt	35	1515 (325)	752 (183)
JLUC224	15	1700*	0.5	Pyp + Phase δ–H + Sti + Melt	66	1311 (515)	483 (206)
SKL420	18	1700	10.5	Pyp + Phase Egg + Sti + Melt	219	788 (96)	403 (53)
JLUC99	18	1900	9	Pyp + Phase δ–H + Sti + Melt	179	510 (68)	261 (27)
JLUZ8	19	1700	36	Pyp + Phase Egg + Sti + Melt	187	615 (45)	272 (20)
JLUC312	20	1700	18	Pyp + Phase δ–H + Sti + Melt	82	728 (77)	422 (87)
S7136	22	1900	12	Pyp + Bdm + Sti + Melt	73	1393 (269)	746 (204)
JLUC361	23	1700	24	Pyp + Phase D + Phase δ–H + Melt	115	847 (135)	427 (106)
S7126	24	1873	12	Pyp + Bdm + Sti + Melt	59	657 (93)	370 (53)
OS3057–ChM	25	1873	0.5	Pyp + Melt	156	1626 (284)	769 (170)
OS3057–ChN	25	1873	0.5	Pyp + Bdm + Melt	157	1496 (173)	739 (144)

* Temperature estimated from heating power. One standard deviation of analyses is given in parentheses. Only small amounts of stishovite appeared in some experiments. Pyp, pyrope; Sti, stishovite; Crn, corundum; Mgs, magnesite; Bdm, bridgmanite.

### FTIR spectra of pyrope

Representative FTIR spectra of synthetic pyrope are displayed in Fig. 1e and Fig. S5. Most FTIR spectra of pyrope garnet synthesized at 5–25 GPa under various high temperatures exhibit a strong, broad and asymmetric absorption peak that is centered at ∼3636 cm^−1^, which is similar to those observed in earlier studies [18–20]. This peak was proposed to correspond to the hydroxyl vibration of $({\mathrm{OH}})_4^{4 - }$ clusters for the substitution of ${\mathrm{SiO}}_4^{4 - }$, i.e. the hydrogarnet substitution, in the pyrope cubic structure [18,19]. However, FTIR spectra of pyrope garnet synthesized at 10–15 GPa and 1100–1700 K (the samples of JLUC268, 236, 244, 277 and 224) exhibit two absorption peaks at ∼3528 and ∼3600 cm^–1^, respectively. Figure S6 shows the FTIR spectra of the representative coexisting phases such as hydrous phase E, stishovite and bridgmanite. These spectra exhibit different absorption bands compared with those of pyrope garnet. The inclusions or contamination effects can also be excluded because we collected FTIR spectra on clean and pure regions in single crystals. These two peaks are close to the previously observed peaks in synthetic pyrope at 6–12 GPa and 1273–1373 K [21,29], Ti-bearing pyrope at 2.5–3 GPa and 1248–1273 K [30], majoritic garnet at 18 GPa and 1773–2073 K [23] and natural pyropic garnet [15,16]. This phenomenon of multiple peaks is possibly related to the weakening of hydrogen bonding and associated proton disorder in the pyrope crystal structure under certain pressure and temperature conditions [19,21].

### Water solubility in synthetic pyrope

We derived the water solubility in pyrope garnet by using the two calibration methods of Bell *et al.* [31] and Paterson [32], respectively (Table 1). The derived values when using the former are generally twice those of the latter. To enable a systematic comparison with previous studies [10,20–25,29,33], we employed the results that were derived from the calibration method of Bell *et al.* [31]. The water solubility in pyrope synthesized at 5–12 GPa and 1273–1473 K, corresponding to conditions of the slab in the upper mantle, ranges from 323 ± 48 to 1622 ± 671 wt. ppm H_2_O. At a constant temperature of 1273 K, the water solubility in pyrope generally increases with increasing pressure up to 12 GPa. These results are consistent with two previous studies [21,29] but contrast with those of Withers *et al.* [20] under similar pressure and temperature conditions. Synthetic pyrope at 14–15 GPa and 1100–1700 K, corresponding to conditions at the topmost of the mantle transition zone, can contain relatively large amounts of water (above 2000 wt. ppm H_2_O). However, the water solubility in pyrope synthesized at 18–24 GPa and 1700–1900 K decreases and remains almost constant with only minor variations (510–1393 wt. ppm H_2_O). With further increasing pressure, the water solubility in pyrope increases to 1626 wt. ppm H_2_O at 25 GPa and 1873 K. These results are comparable to those of pyrope-rich garnet synthesized from a basaltic composition at pressures of 20 and 25 GPa and temperatures of 1673–2023 K [22,24], although the compositions differ somewhat between the present and previous studies. The elevated water solubility in pyrope at 25 GPa may be attributed to the resealed water from the decomposition of the hydrous phases of phase D or phase δ–H, which increases the local water activity and thus enhances water incorporation into the pyrope [34].

## DISCUSSION

### Water solubility in pyrope versus pressure and temperature

**Figure 2.**
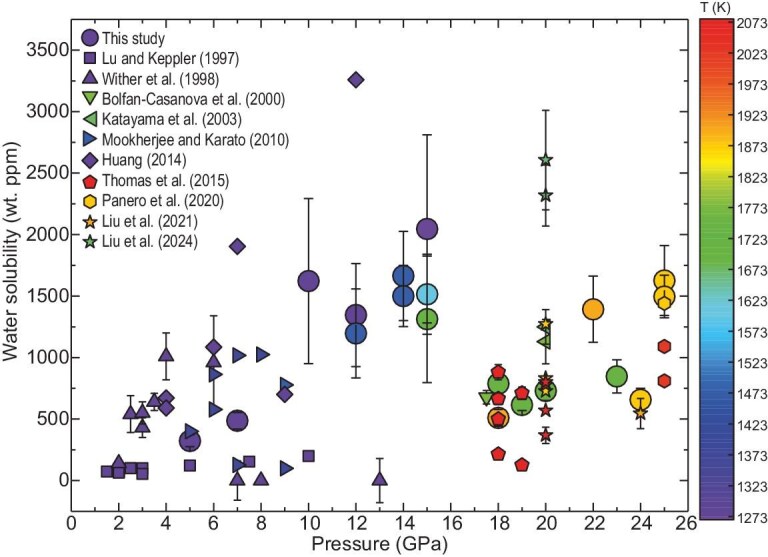
Water solubility in pyrope in our and previous studies under various pressure and temperature conditions. Circle symbols represent our study, while other shapes of the symbols represent previous studies (square, Lu and Keppler, 1997 [10]; triangle, Wither *et al.*, 1998 [20]; inverted triangle, Bolfan-Casanova *et al.*, 2000 [56]; left triangle, Katayama *et al.*, 2003 [22]; right triangle, Mookherjee and Karato, 2010 [21]; diamond, Huang, 2014 [29]; pentagon, Thomas *et al.*, 2015 [23]; hexagon, Panero *et al.*, 2020 [24]; star, Liu *et al.*, 2021 (24 GPa) [33] and Liu *et al.*, 2024 (20 GPa) [25]). The symbols are colored for temperature according to the scale bar.

Although garnets in basaltic slabs also contain Fe, Ca and other cations, we focus on pure pyrope garnet with a relatively simple chemical composition in the present study to demonstrate the dependence of water solubility on pressure and temperature in the deep mantle. As shown in Table 1 and Fig. 2, water solubility in pyrope is significantly affected by either pressure or temperature. To clarify these relationships, we modified the following thermodynamic model that is widely used to constrain water solubility in nominally anhydrous minerals [26,35,36]:


(1) \begin{eqnarray*}
{c}_{{\mathrm{water}}}\ = \ f_{{\rm H}_2{\rm O}}^n \cdot {\mathrm{exp}}\left( { - \frac{{\Delta H - T\Delta S + P\Delta V}}{{{\mathrm{R}}T}}} \right),\end{eqnarray*}

where${\mathrm{\ }}{c}_{{\mathrm{water}}}$ is the water solubility in minerals; ${f}_{{{\mathrm{H}}}_2{\mathrm{O}}}$ is the water fugacity; *n* is the fugacity exponent; ${\mathrm{\Delta }}H$ and ${\mathrm{\Delta }}S{\mathrm{\ }}$are the reaction enthalpy and entropy, respectively; ${\mathrm{\Delta }}V$ is the volume change of the solids; *P* is the pressure; *T* is the temperature; and R is the gas constant. This equation can be expressed as a logarithmic function:


(2) \begin{eqnarray*}
{\mathrm{ln}}{c}_{{\mathrm{water}}}{\mathrm{\ }} = {\mathrm{\ }}n \cdot {\mathrm{ln}}{f}_{{{\mathrm{H}}}_2{\mathrm{O}}}{\mathrm{\ }} + {\mathrm{\ }}\frac{{{\mathrm{\Delta }}S}}{{\mathrm{R}}}{\mathrm{\ }} - {\mathrm{\ }}\frac{{{\mathrm{\Delta }}H{\mathrm{\ }} + {\mathrm{\ \Delta }}V \cdot P}}{{{\mathrm{R}}T}}.
\end{eqnarray*}

It should be noted that the equation of state of pure H_2_O cannot be used for determining water fugacity in silicate-bearing systems under HPHT conditions, in which the complete miscibility between aqueous fluids and silicate melts occurs at >1.5 GPa [37]. Experimental studies have revealed that dissolved water concentrations in such silicate melts possibly exhibit some pressure–temperature dependencies [38–40]. Nevertheless, quantitative constraints on water fugacity under deep-mantle conditions, particularly across the mantle transition zone to the uppermost lower mantle, remain poorly constrained. Dong *et al.* [35] further demonstrated that the enhanced fluid–melt miscibility under HPHT conditions may explain the observed statistical insignificance of the water fugacity term in thermodynamic models for olivine, wadsleyite and ringwoodite. Therefore, the water solubility in nominally anhydrous minerals can be simplified as:


(3) \begin{eqnarray*}
{\mathrm{ln}}{c}_{{\mathrm{water}}}{\mathrm{\ }} = {\mathrm{\ }}A{\mathrm{\ }} + {\mathrm{\ }}\frac{{B{\mathrm{\ }} + {\mathrm{\ }}C \times P}}{T},
\end{eqnarray*}

where *A, B* and *C* are parameters related to the changes in entropy, enthalpy and volume of the hydration reaction, respectively. Such a simplified equation under constant pressure is also adopted in previous studies on the water solubility in nominally anhydrous minerals [25,35].

We applied a non-linear least-squares regression to optimize the parameters of Equation (3) by using pyrope garnet water-solubility data from the present and previous studies (Fig. 2) and obtained the following parameters: *A* = 3.42 ± 0.85, *B* = 2126.17 ± 995.27 and *C* = 213.82 ± 31.64. The regression yielded a moderate coefficient of determination (*R*² = 0.49), which primarily reflects inherent data scatter and inter-experimental uncertainties across the compiled datasets. To evaluate the robustness of the above fitting, we also performed comparative regression analyses by using both linear and second-order polynomial functions of pressure and temperature. We found that fitting the current data by using Equation (3) produces a better value of *R*² than those produced by using either linear (*R*² = 0.40) or second-order (*R*² = 0.47) polynomial functions. Thus, the optimized solubility model demonstrates robust consistency with experimental data, as shown in Fig. S7, despite challenges in quantifying water fugacity within hydrous silicate melts under HPHT conditions.

**Figure 3.**
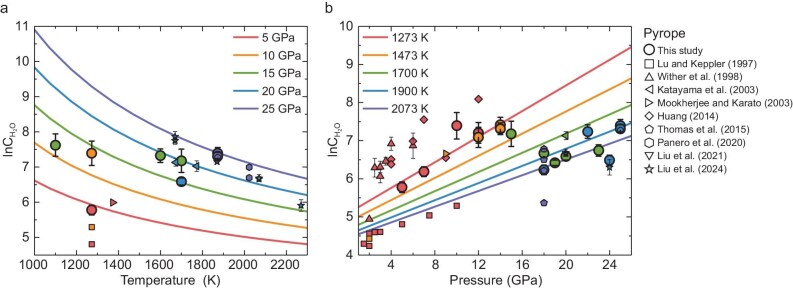
Water solubility in pyrope as a function of (a) temperature and (b) pressure. The curves are the fitting results by using Equation (3). Circle symbols represent the present study, while other shapes of the symbols represent previous studies (square, Lu and Keppler, 1997 [10]; triangle, Wither *et al.*, 1998 [20]; left triangle, Katayama *et al.*, 2003 [22]; right triangle, Mookherjee and Karato, 2010 [21]; diamond, Huang, 2014 [29]; pentagon, Thomas *et al.*, 2015 [23]; hexagon, Panero *et al.*, 2020 [24]; inverted triangle, Liu *et al.*, 2021 [33]; star, Liu *et al.*, 2024 [25]). The colors of the symbols represent different pressures and temperatures, respectively.

We employed these derived thermodynamic parameters to calculate the water solubility in pyrope garnet by using a natural logarithmic function across a temperature range of 1000–2300 K and pressures of 1–26 GPa, as shown in Fig. 3. In addition, Figs S8 and S9 display the calculated curves with uncertainties at HPHT and all the data fall within our calculated uncertainties. The water solubility in pyrope garnet exhibits a negative temperature dependence at constant pressure whereas it shows a positive relationship with pressure at constant temperature. The inverse correlation between temperature and pressure that governs water content results in a solubility maximum, which accounts for the observed peak in water solubility in pyrope at 15 GPa in our experiments (Table 1). At constant pressure, the decrease in water solubility with increasing temperature arises from reduced water content in silicate melts, driven by an increase in the melt fraction at elevated temperatures [40]. This trend aligns with observations in most mantle minerals, such as olivine, wadsleyite, ringwoodite, majorite and stishovite [25,35,41]. Conversely, pressure may enhance water solubility, likely through increased water activity in silicate melts [42].

### Hydrogen-incorporation mechanisms in pyrope garnet

The primary hydrogen-incorporation mechanisms in pyrope garnet are the hydrogen substitution of Mg in dodecahedral sites (H^+^ ↔ 2Mg^2+^) or Si in tetrahedral sites (4H^+^ ↔ Si^4+^ or H^+^ + Al^3+^ ↔ Si^4+^) [10,21,22,43]. As shown in Fig. S10, there is no systematic correlation between the water solubility and the aluminum content in these majoritic and pyrope garnets [21–25]. This fact suggests that hydrogen is more likely to be incorporated into the Mg and Si sites rather than into the Al-related substitution sites within the garnet structure. Otherwise, the water solubility of pyrope garnet would show a significantly higher water content than these majoritic garnets with lower alumina contents. First-principles calculations also suggested that hydrogen substitution at Si and Mg sites is energetically more favorable than the substitution at Al sites because the formation energy of the former is lower than that of the latter [43]. FTIR spectra in this study and prior work reveal a prominent absorption peak at 3630 cm^–1^ that is associated with the hydrogarnet substitution (4H^+^ ↔ Si^4+^) [20,25,29]. However, the presence of multiple IR peaks in our study implies that additional substitution mechanisms may coexist in pyrope. To resolve these complexities, advanced characterization techniques such as solid-state ^1^H nuclear magnetic resonance or neutron scattering should be employed to elucidate the hydrogen-incorporation pathways in pyrope garnet.

### Geophysical implications

**Figure 4.**
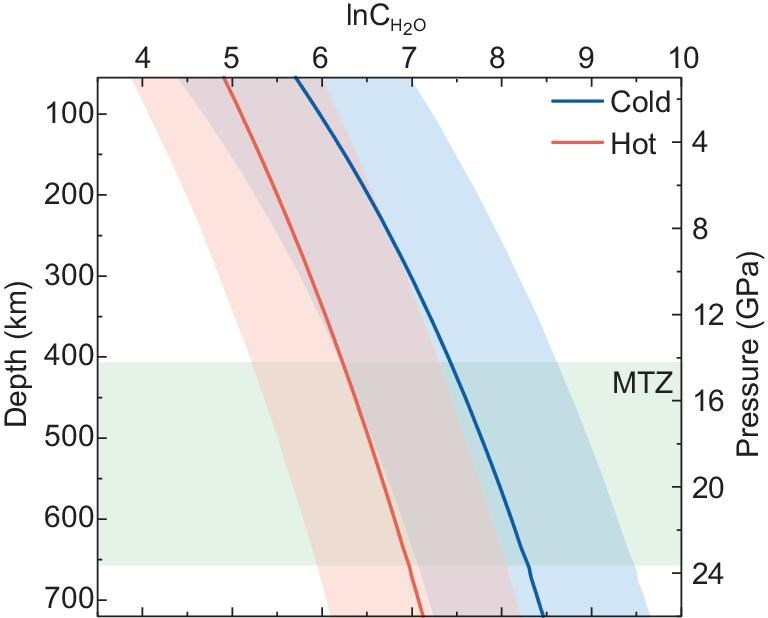
Water solubility in pyrope garnet along cold (blue line) and hot (red line) subducted slabs, respectively. The shaded regions represent the uncertainties for water solubility. The subduction geotherm gradient that is used in the calculations is from Ohtani *et al.* [44] and Katsura [45].

We further assessed the variations in the water-storage capacity of pyrope garnet along both cold and hot subducting slab geotherms [44,45]. As shown in Fig. 4, the water solubility in pyrope increases with depth along a cold slab geotherm, reaching a maximum of ∼0.47 wt% H_2_O at the topmost lower mantle (720 km), prior to pyrope decomposition. In contrast, along a hot subduction geotherm, the solubility follows a similar trend but with lower values, reaching a maximum of ∼0.12 wt% H_2_O at the same depth. Therefore, pyrope garnet is one of the primary hosts and carriers for water in the deep mantle of Earth and may dominate the water budget of subducted basaltic slabs.

Some natural pyrope-rich garnets of mantle origin contain up to 2200 wt. ppm of water, although most garnets incorporate <500 wt. ppm of water [13–17]. The water solubility in these natural garnets represents a lower limit owing to the different water fugacity environments or potential water loss during ascent in different regions. Our experiments, in conjunction with earlier studies, are conducted under water-saturated environments and the findings represent the maximum water solubility in pyrope garnets. Thus, the variability of water solubility in natural pyrope can be explained by our results.

As the oceanic crust subducts into the deep mantle, especially at the base of the mantle transition zone, hydrous phases in subducting slabs, such as superhydrous phase B and hydrous ringwoodite, would release water to form hydrous melts. This occurs because the nearly-dry phases of bridgmanite and periclase that are prevalent in these regions cannot retain any amounts of water [33,46]. The high water-storage capacity of pyrope-rich garnet, together with stishovite in the mantle transition zone and the topmost lower mantle, enables the retention of water that is released from the aforementioned hydrous phases or silicate melts until the pyrope-rich garnet is saturated with water. At depths below 720 km, water from hydrous melts produced by the dehydration of pyrope garnet into dry bridgmanite plus corundum will be further stored in stishovite [47]. Consequently, the hydrated pyrope-rich garnet may prevent the occurrence of partial melting of subducted slabs at the topmost lower mantle.

The incorporation of a substantial quantity of water would dramatically affect the physical and chemical properties of pyrope-rich garnet and thereby the subducting slabs. Water has a notable influence on the mechanical strength of pyrope-rich garnet [48]. The substantial amount of water in pyrope-rich garnet may result in subducting basaltic slabs that are weaker than the surrounding mantle at depths from the mantle transition zone to the topmost lower mantle. Water may also affect the post-garnet-phase boundary from pyrope to bridgmanite plus corundum at the topmost lower mantle, which may alter its impact on the slab stagnation at depths of 660–1000 km [12]. Such significant water may shift the garnet–bridgmanite phase boundary toward lower pressures by 2 GPa compared with dry conditions [49]. Furthermore, water also enhances the electrical conductivity of pyrope-rich garnet [50]. The presence of hydrated pyrope-rich garnet may contribute to the observed high electrical conductivity anomalies along the circum-Pacific and western North American subducting slabs in the topmost lower mantle [51,52].

## METHODS

### Starting materials

We used two oxide mixtures for the starting materials. One was a pyrope composition of Mg_3_Al_2_Si_3_O_12_ plus 8.8 wt% H_2_O and the other was Mg_0.9_Al_0.2_Si_0.9_O_3_ plus 8.8 wt% H_2_O (S7126 and S7136). The water was induced by Mg(OH)_2_. The oxide mixtures of SiO_2_, Al_2_O_3_, MgO and Mg(OH)_2_ were ground in an agate mortar for 1 h. The mixtures were dried at 100°C for 24 h prior to experiments.

### Synthesis experiments

HPHT synthesis experiments were conducted at pressures of 5−25 GPa and temperatures of 1100−1900 K by using 1000-ton Walker-type and 1200-ton large-volume presses at the State Key Laboratory of High Pressure and Superhard Materials, Jilin University and the Bayerisches Geoinstitut, Bayreuth University (S7126 and S7136), respectively. Pressure calibration was established in earlier studies [53,54]. The starting materials were sealed into Pt_90_Rh_10_ capsules and then loaded into the experimental cell assemblies. We used semi-sintered magnesia octahedra with edge lengths of 14, 10 and 8 mm together with tungsten carbide anvils with truncation edge lengths of 8, 5 and 3 mm. The temperature was monitored by using a D-type (W_97_Re_3_/W_75_Re_25_) thermocouple adjacent to the base of the capsules. The cell assembly was first compressed to the desired pressure at room temperature and then heated to the target temperature at a rate of 50°C/min. To grow large-sized and high-quality single crystals, the target temperature and pressure were maintained for 0.5–40 h. The samples were quenched to room temperature by turning off the heating power and were then gradually decompressed to ambient pressure over 24 h. Single crystals of pyrope garnet in HPHT runs of OS3033-ChM, OS3033-ChN, OS3057-ChM and OS3057-ChN by Xu *et al.* [55] were used to further explore the water solubility in pyrope.

### Sample characterization

Recovered samples were polished with abrasive paper and diamond pastes for characterization. The textures of the samples were observed by using a Magellan 400 field emission scanning electron microscope equipped with a backscattered electron detector. Phases in the samples were identified by using a Renishaw Raman spectrometer equipped with a 532-nm laser source. The chemical compositions of the phases in the samples were identified by using a JEOL JXA-8230 electron probe microanalyser with a wavelength dispersive spectrometer. Forsterite served as a standard for Mg and Si, and pyrope for Al.

### Water-solubility calculation

The water solubility in the pyrope garnet was calculated based on the hydroxyl peaks observed from the FTIR measurements. The run products were polished on both sides to a thickness of 35–220 μm based on the grain size of the single crystals for the FTIR measurements. Spectra were obtained with 512 scans, 4 cm^–1^ resolution and apertures of 40–100 μm by using a Bruker Vertex 80V spectrometer. More than 10 spectra were collected from each sample for FTIR spectral analysis. The water solubility of the pyrope was calculated by using the Beer–Lambert law:


(4) \begin{eqnarray*}
c = \frac{1}{I}\smallint K\left( {\bar{\nu }} \right){\mathrm{d}}\bar{\nu },
\end{eqnarray*}

where *c* is the concentration of the water in mol/l, *I* is the integral molar absorption coefficient in l·cm^−2^·mol^−1^ and $K( {\bar{\nu }} )$ is the absorption coefficient in cm^–1^ as a function of wave number $\bar{\nu }$. To express the water solubility as wt. ppm, *c* was multiplied by a density factor ${X}_i$: ${X}_i = {10}^6 \times ( {18/2d} )$, where *d* is the mineral density in g/l and taken as 3560 g/l in our study [56].

## Supplementary Material

nwaf133_Supplemental_File
