# Supplementary material for: The role of pyrope garnet in water transport into the topmost lower mantle
Source: Natl Sci Rev. 2025 Apr 4;12(6):nwaf133. doi: 10.1093/nsr/nwaf133 (PMC12063092; doi:10.1093/nsr/nwaf133)
Supplement: nwaf133_Supplemental_File [file nwaf133_supplemental_file.docx]

**Supporting Information for**

The Role of Pyrope Garnet in Water Transport into the Topmost Lower Mantle

Luyao Chen^1,†^, Xinyu Zhao^1,†^, Chaowen Xu^2^, Xin Li^1^, Xinran Zhang^3^, Bingtao Feng^1^, Kuo Hu^1,*^, Bingbing Liu^1^, Zhaodong Liu^1,^^4,*^_，_Wenliang Xu^4,*^

^1^State Key Laboratory of High Pressure and Superhard Materials, Synergetic Extreme Condition User Facility, Jilin University, Changchun, 130012, China.

^2^Institute of Earthquake Forecasting, China Earthquake Administration, Beijing, 100036, China.

^3^College of Chemistry, Jilin University, Changchun, 130012, China.

^4^College of Earth Sciences, Jilin University, Changchun, 130061, China.

^*^Corresponding author: Zhaodong Liu, Kuo Hu, Wenliang Xu

**Email:** liu_zhaodong@jlu.edu.cn, hukuo@jlu.edu.cn, xuwl@jlu.edu.cn

^†^L.C. and X.Z. contributed equally to this work.


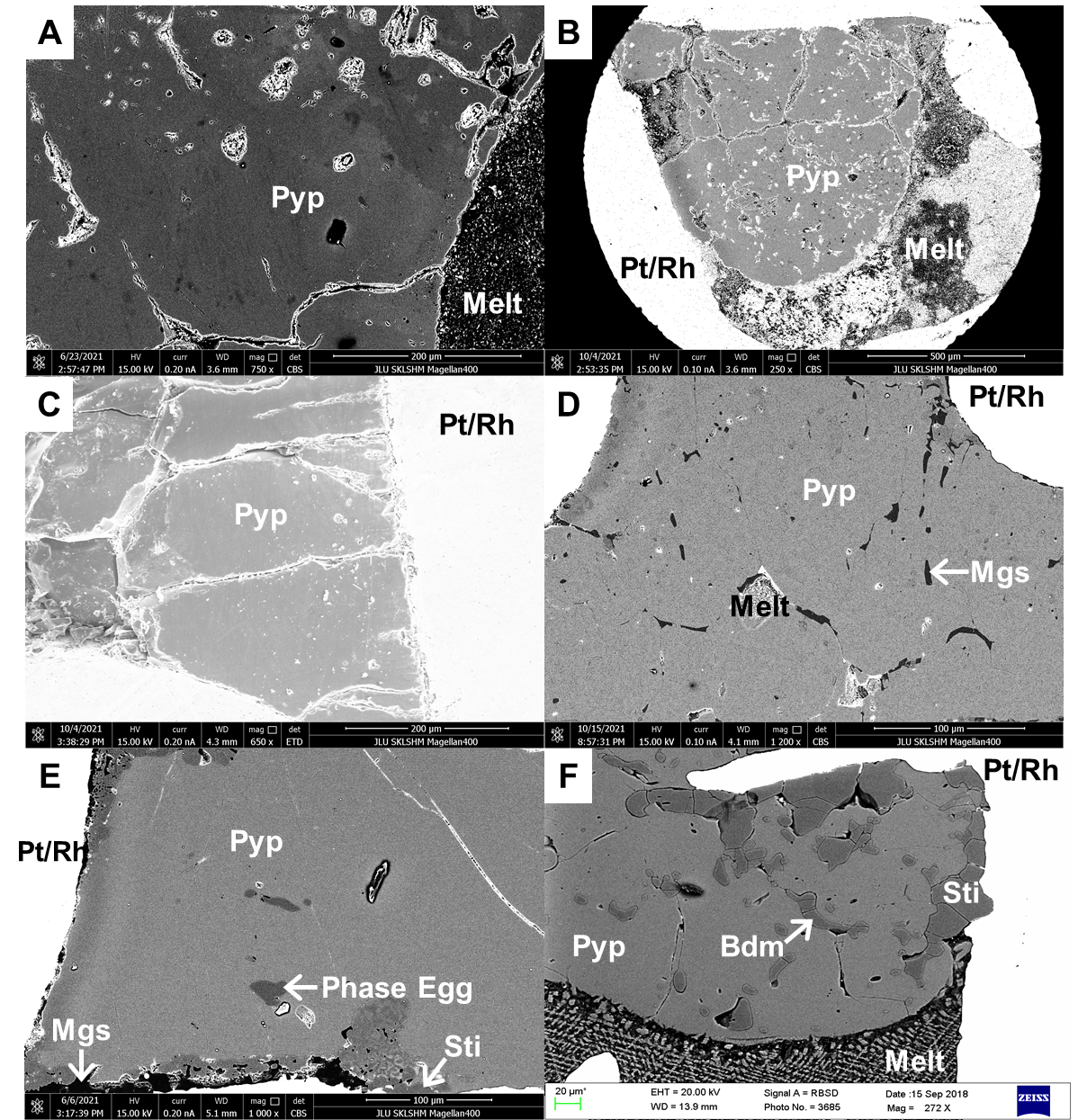


Figure S1. Back-scattered electron images of the recovered samples: (A) JLUC230 (5 GPa and 1273 K); (B) JLUC268 (10 GPa and 1273 K); (C) JLUC244 (12 GPa and 1473 K); (D) JLUC277 (15 GPa and 1100 K); (E) JLUZ8 (19 GPa and 1700 K); (F) S7126 (24 GPa and 1873 K). Abbreviations: Pyp, pyrope; Mgs, magnesite; Sti, stishovite; Bdm, bridgmanite.


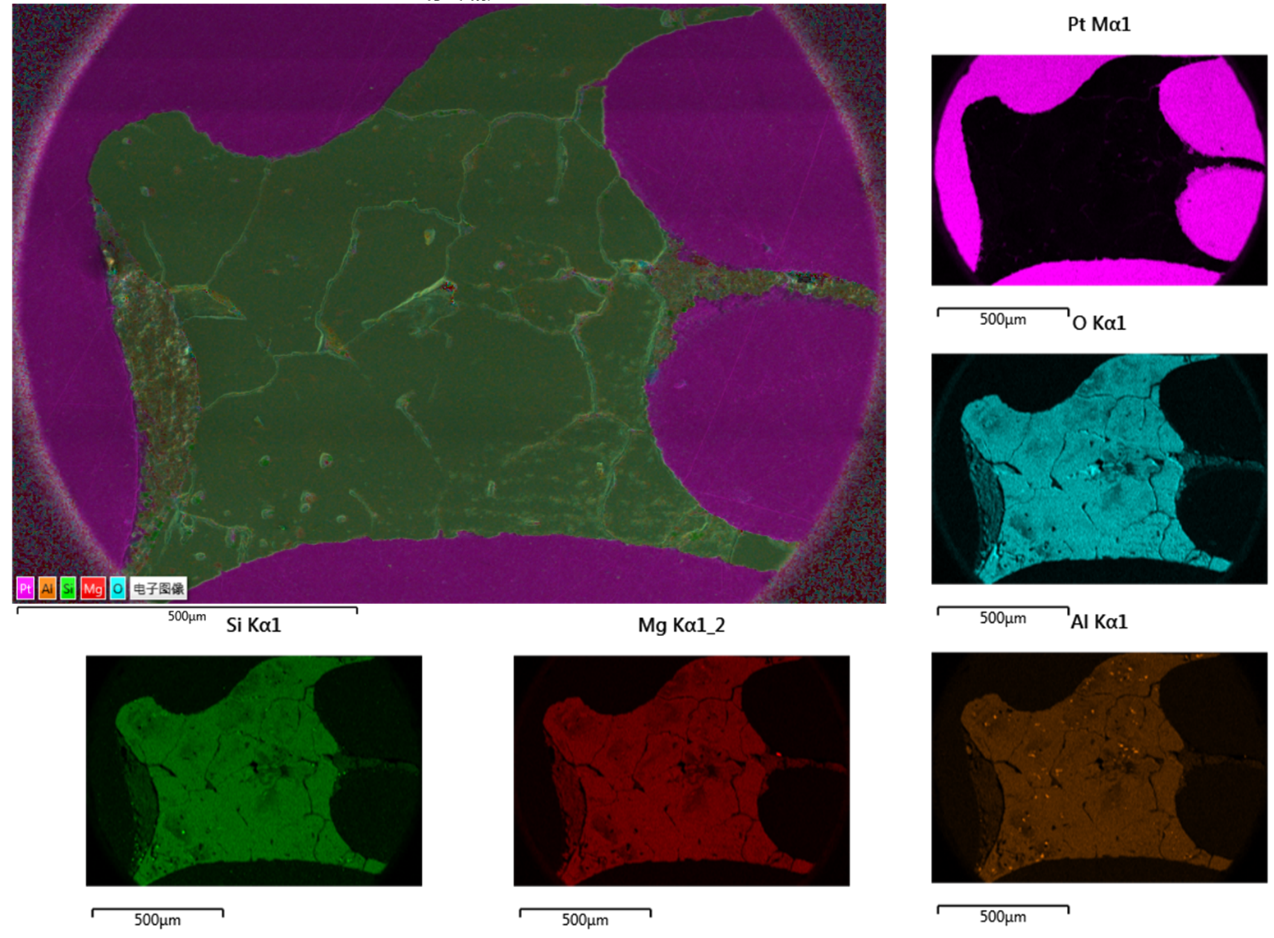


Figure S2. Element mapping of the recovered sample JLUC230 (5 GPa and 1273 K).


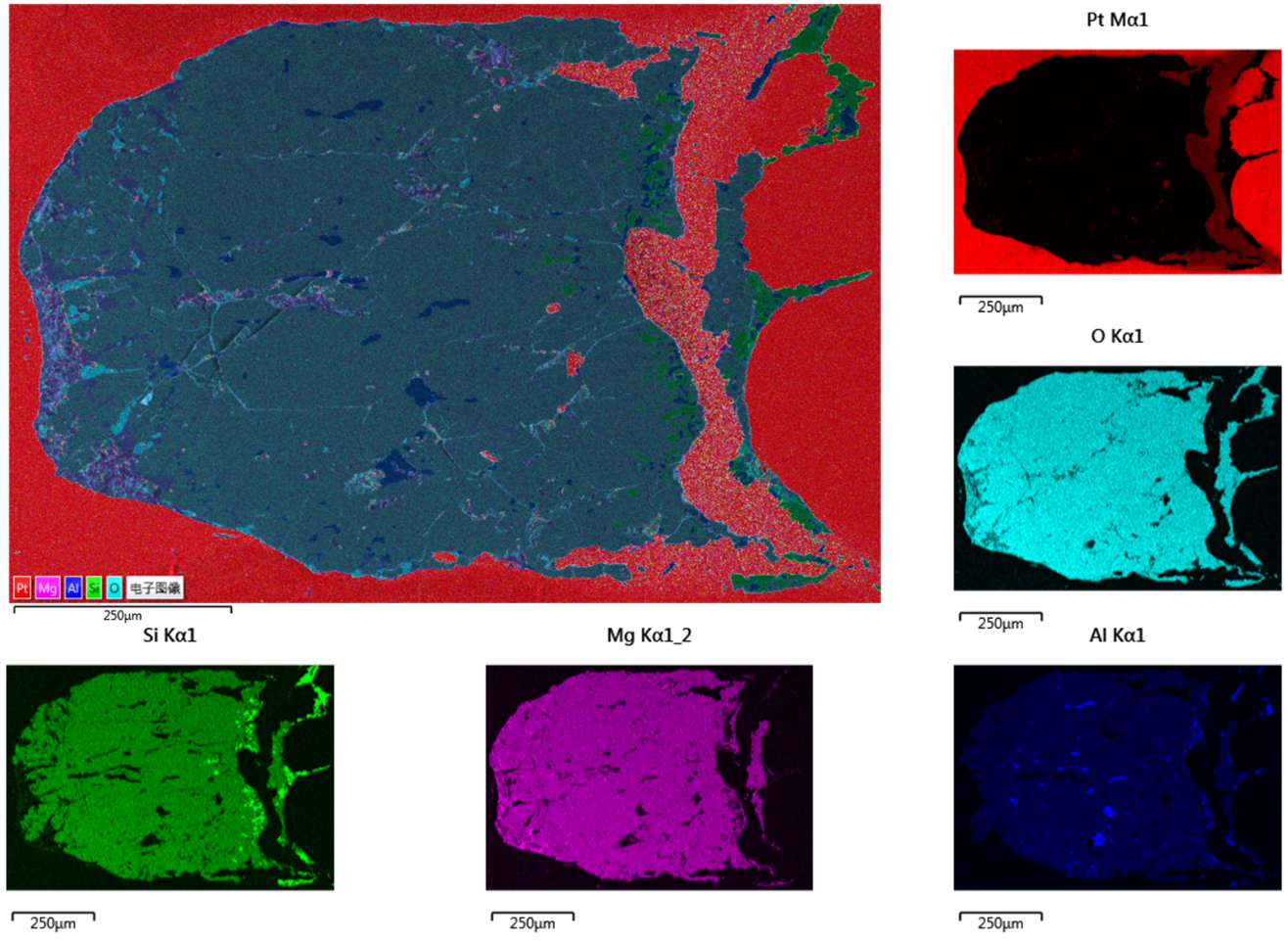


Figure S3. Element mapping of the recovered sample JLUC99 (18 GPa and 1900 K).





Figure S4. Representative Raman spectra of pyrope for various experimental runs (JLUC268, 10 GPa and 1273 K; JLUC224, 15 GPa and 1700 K; JLUZ8, 19 GPa and 1700 K; S7126, 24 GPa and 1873 K). Spectra are offset vertically for clarity.





Figure S5. Representative FTIR spectra of pyrope synthesized at different pressures and temperatures (JLUC305, 7 GPa and 1273 K; JLUC312, 20 GPa and 1700 K; S7126, 24 GPa and 1873 K; JLUC99, 18 GPa and 1900 K; SKL420, 18 GPa and 1700 K; JLUC361, 23 GPa and 1700 K; JLUC364, 15 GPa and 1600 K; OS3033–ChM, 14 GPa and 1473 K; OS3057–ChN, 25 GPa and 1873 K; JLUC224, 15 GPa and 1700 K; JLUC268, 10 GPa and 1273 K; JLUC236, 12 GPa and 1273 K; JLUC244, 12 GPa and 1473 K). Spectra are offset vertically for clarity.





Figure S6. Representative FTIR spectra of hydrous phases, stishovite, and bridgmanite synthesized at different pressures and temperatures (OS3057–ChN, 25 GPa and 1873 K; JLUC312, 20 GPa and 1700 K; OS3033–ChM, 14 GPa and 1473 K; OS3033–ChN, 14 GPa and 1473 K). Spectra are offset vertically for clarity.





Figure S7. Comparison of experimental versus calculated water content in pyrope garnet. The dashed line represents the linear regression between the experimental measurements and the theoretical estimates of water content (R² = 0.49). Light and dark red shaded regions indicate 95% confidence intervals and prediction intervals, respectively. Symbols represent experimental data: circle (this study), square (Lu & Keppler, 1997 [1]), triangle (Withers et al., 1998 [2]), left triangle (Katayama et al., 2003 [3]), right triangle (Mookherjee and Karato, 2010 [4]), diamond (Huang, 2014 [5]), pentagon (Thomas et al., 2015 [6]), hexagon (Panero et al., 2020 [7]), inverted triangle (Liu et al., 2021 [8]), and star (Liu et al., 2024 [9]).





Figure S8. Water solubility in pyrope as a function of temperature at constant pressures (5–25 GPa). The curves represent the fitted results, and the shaded areas represent their uncertainties. Symbols represent experimental data: circle (this study), square (Lu & Keppler, 1997 [1]), triangle (Withers et al., 1998 [2]), left triangle (Katayama et al., 2003 [3]), right triangle (Mookherjee and Karato, 2010 [4]), diamond (Huang, 2014 [5]), pentagon (Thomas et al., 2015 [6]), hexagon (Panero et al., 2020 [7]), inverted triangle (Liu et al., 2021 [8]), and star (Liu et al., 2024 [9]). The color of the curves and symbols corresponds to the pressure (5–25 GPa).





Figure S9. Water solubility in pyrope as a function of pressure at fixed temperatures (1273–2073 K). The curves represent the fitted results, and the shaded areas represent their uncertainties. Symbols represent experimental data: circle (this study), square (Lu & Keppler, 1997 [1]), triangle (Withers et al., 1998 [2]), left triangle (Katayama et al., 2003 [3]), right triangle (Mookherjee and Karato, 2010 [4]), diamond (Huang, 2014 [5]), pentagon (Thomas et al., 2015 [6]), hexagon (Panero et al., 2020 [7]), inverted triangle (Liu et al., 2021 [8]), and star (Liu et al., 2024 [9]). The color of the curves and symbols corresponds to the temperature (1273–2073 K).





Figure S10. Aluminum content dependence of water solubility in pyrope-rich garnet. Circle symbols represent the present study, and other shapes of the symbols represent earlier studies (square, Katayama et al., 2003 [3]; diamond, Mookherjee and Karato, 2010 [4]; pentagon, Thomas et al., 2015 [6]; hexagon, Panero et al., 2020 [7]; star, Liu et al., 2024 [9]).

Table S1 Chemical compositions of recovered samples determined by electron microprobe.

| **Run No.** | **Phases** | **MgO** | **Al_2_O_3_** | **SiO_2_** | **Total** | **Mg** | **Al** | **Si** | **O** |
| --- | --- | --- | --- | --- | --- | --- | --- | --- | --- |
| JLUC230 | Pyrope (n=6) | 30.13 (0.41) | 24.95 (0.46) | 44.83 (0.84) | 99.92 (1.02) | 3.016 (0.024) | 1.975 (0.044) | 3.011 (0.032) | 12 |
|  | Melt (n=5) | 27.67 (1.39) | 14.92 (3.72) | 24.20 (3.87) | 66.79 (8.96) | 0.717 (0.070) | 0.300 (0.036) | 0.416 (0.008) | 2 |
| JLUC305 | Pyrope (n=8) | 30.81 (0.24) | 23.18 (0.39) | 45.59 (0.30) | 99.59 (0.57) | 3.095 (0.024) | 1.841 (0.025) | 3.072 (0.015) | 12 |
|  | Phase Egg (n=1) | 0.15 | 62.53 | 31.61 | 94.29 | 0.003 | 0.847 | 0.363 | 4 |
|  | Melt (n=7) | 9.43 (1.70) | 10.03 (8.29) | 28.11 (13.38) | 47.57 (20.00) | 0.398 (0.278) | 0.228 (0.147) | 0.630 (0.073) | 2 |
| JLUC268 | Pyrope (n=7) | 30.63 (0.19) | 24.76 (0.75) | 45.57 (0.65) | 100.96 (0.32) | 3.034 (0.015) | 1.939 (0.058) | 3.028 (0.043) | 12 |
|  | Melt (n=8) | 1.67 (1.00) | 27.34 (12.08) | 0.55 (0.12) | 29.56 (11.77) | 0.111 (0.068) | 1.229 (0.050) | 0.023 (0.006) | 2 |
| JLUC236 | Pyrope (n=6) | 30.80 (0.23) | 23.61 (1.15) | 45.93 (0.58) | 100.35 (0.50) | 3.069 (0.030) | 1.860 (0.084) | 3.070 (0.049) | 12 |
|  | Melt (n=7) | 24.11 (1.89) | 18.29 (1.39) | 39.03 (3.99) | 81.42 (4.24) | 0.491 (0.030) | 0.296 (0.031) | 0.533 (0.034) | 2 |
| JLUC244 | Pyrope (n=6) | 30.41 (0.29) | 25.35 (0.40) | 44.86 (0.18) | 100.63 (0.63) | 3.024 (0.021) | 1.993 (0.022) | 2.993 (0.016) | 12 |
|  | Melt (n=11) | 13.03 (13.97) | 28.90 (26.21) | 41.71 (24.88) | 83.64 (20.20) | 0.351 (0.503) | 0.425 (0.383) | 0.506 (0.267) | 2 |
| JLUC277 | Pyrope (n=7) | 29.86 (0.62) | 24.84 (0.67) | 45.19 (0.46) | 99.89 (0.44) | 2.987 (0.012) | 1.965 (0.049) | 3.033 (0.034) | 12 |
|  | Stishovite (n=5) | 0.04 (0.07) | 2.03 (0.91) | 95.77 (0.99) | 97.84 (0.80) | 0.001 (0.001) | 0.024 (0.011) | 0.981 (0.008) | 2 |
|  | Phase Egg (n=4) | 0.07 (0.01) | 54.17 (1.47) | 33.64 (1.76) | 87.87 (2.25) | 0.002 (0.001) | 1.566 (0.038) | 0.825 (0.029) | 4 |
|  | Corundum (n=4) | 0.78 (0.05) | 61.90 (0.32) | 37.92 (0.42) | 100.59 (0.16) | 0.019 (0.001) | 1.174 (0.007) | 0.610 (0.006) | 3 |
|  | Melt (n=2) | 24.60 (2.19) | 21.54 (1.85) | 34.39 (2.53) | 80.53 (6.57) | 0.511 (0.004) | 0.354 (0.002) | 0.479 (0.003) | 2 |
|  | Magnesite (n=2) | 39.94 (0.11) | 0.04 (0.05) | 0.03 (0.02) | 40.02 (0.15) | 0.998 (0.001) | 0.001 (0.0003) | 0.001 (0.001) | 1 |
| JLUC364 | Pyrope (n=33) | 30.12 (0.21) | 24.75 (0.34) | 44.53 (0.37) | 99.40 (0.38) | 3.032 (0.020) | 1.970 (0.027) | 3.007 (0.020) | 12 |
|  | Superhydrous phase B (n=1) | 54.516 | 16.088 | 19.861 | 90.465 | 8.70170125 | 2.030141472 | 2.126543271 | 16 |
|  | Corundum (n=4) | 0.79 (0.16) | 97.20 (0.62) | 1.19 (0.25) | 99.19 (0.28) | 0.020 (0.004) | 1.959 (0.008) | 0.020 (0.004) | 3 |
|  | Melt (n=8) | 10.45 (3.62) | 12.10 (2.95) | 20.39 (5.96) | 42.95 (12.43) | 0.395 (0.028) | 0.371 (0.028) | 0.524 (0.018) | 2 |
| JLUC224 | Pyrope (n=4) | 30.41 (0.52) | 24.29 (1.11) | 44.25 (0.56) | 98.95 (0.31) | 3.077 (0.060) | 1.943 (0.085) | 3.004 (0.039) | 12 |
|  | Phase Egg (n=5) | 1.10 (0.10) | 41.33 (1.56) | 50.43 (3.69) | 92.86 (2.06) | 0.037 (0.004) | 1.111 (0.070) | 1.148 (0.055) | 4 |
| SKL420 | Pyrope (n=6) | 29.70 (0.22) | 25.86 (0.44) | 43.97 (0.45) | 99.53 (0.74) | 2.987 (0.024) | 2.055 (0.030) | 2.965 (0.015) | 12 |
|  | Phase Egg (n=1) | 0.36 | 45.00 | 53.21 | 98.57 | 0.012 | 1.137 | 1.141 | 4 |
| JLUC99 | Pyrope (n=10) | 30.13 (0.59) | 23.89 (0.26) | 44.20 (0.81) | 98.22 (1.54) | 3.070 (0.017) | 1.925 (0.023) | 3.021 (0.013) | 12 |
|  | Melt (n=2) | 30.39 | 2.99 | 9.69 | 43.07 | 1.293 | 0.101 | 0.278 | 2 |
| JLUZ8 | Pyrope (n=6) | 31.09 (0.30) | 23.42 (0.15) | 45.18 (0.12) | 99.69 (0.33) | 3.123 (0.023) | 1.860 (0.011) | 3.044 (0.013) | 12 |
|  | Phase Egg (n=2) | 0.18 (0.02) | 37.89 (0.72) | 49.99 (0.03) | 88.09 (0.71) | 0.006 (0.001) | 1.068 (0.013) | 1.196 (0.009) | 4 |
|  | Stishovite (n=2) | 0.01 (0.01) | 2.29 (0.38) | 95.17 (0.00) | 97.47 (0.39) | 0 | 0.028 (0.005) | 0.979 (0.003) | 2 |
|  | Magnesite (n=2) | 40.33 (1.37) | 0.02 (0.004) | 0 | 40.45 (1.27) | 0.999 (0.0001) | 0 | 0 | 1 |
| JLUC312 | Pyrope (n=8) | 30.18 (0.66) | 24.91 (1.32) | 43.99 (0.53) | 99.08 (0.82) | 3.050 (0.071) | 1.990 (0.095) | 2.983 (0.044) | 12 |
|  | Stishovite (n=5) | 0.01 (0.02) | 3.86 (0.08) | 93.81 (0.92) | 97.68 (0.85) | 0 | 0.047 (0.001) | 0.965 (0.001) | 2 |
|  | Phase δ-H (n=5) | 2.81 (0.31) | 84.85 (1.52) | 4.72 (0.70) | 92.38 (0.66) | 0.051 (0.006) | 1.222 (0.015) | 0.058 (0.009) | 2 |
|  | Corundum (n=2) | 2.65 (0.09) | 91.67 (0.03) | 4.83 (0.35) | 99.15 (0.40) | 0.067 (0.002) | 1.845 (0.009) | 0.082 (0.006) | 3 |
|  | Phase D (n=1) | 20.96 | 17.91 | 40.13 | 79.01 | 1.31 | 0.885 | 1.682 | 6 |
|  | Melt (n=3) | 27.49 | 11.08 | 12.58 | 51.15 | 1.068 | 0.259 | 0.272 | 2 |
| S7136 | Pyrope (n=5) | 30.97 (0.61) | 21.31 (1.43) | 46.10 (0.89) | 98.38 (0.52) | 3.150 (0.074) | 1.719 (0.110) | 3.136 (0.052) | 12 |
|  | Stishovite (n=5) | 0 | 2.25 (0.07) | 96.78 (0.48) | 99.02 (0.53) | 0 | 0.027 (0.001) | 0.980 (0.001) | 2 |
|  | Bridgmanite (n=5) | 40.38 (0.47) | 2.19 (0.35) | 58.69 (0.59) | 101.26 (0.75) | 0.995 (0.015) | 0.043 (0.006) | 0.970 (0.006) | 3 |
|  | Melt (n=5) | 30.28 (0.66) | 12.33 (0.53) | 41.42 (0.75) | 84.03 (1.38) | 0.603 | 0.194 | 0.553 | 2 |
| JLUC361 | Pyrope (n=7) | 30.89 (0.90) | 23.04 (0.97) | 44.60 (0.48) | 98.53 (1.45) | 3.140 (0.070) | 1.851 (0.070) | 3.042 (0.027) | 12 |
|  | Phase δ-H (n=7) | 6.56 (0.76) | 60.38 (2.94) | 12.76 (1.91) | 79.70 (1.33) | 0.138 (0.017) | 1.002 (0.045) | 0.180 (0.027) | 2 |
|  | Phase D (n=4) | 20.61 (0.92) | 23.22 (1.57) | 40.55 (1.45) | 84.38 (1.08) | 1.205 (0.042) | 1.074 (0.070) | 1.592 (0.064) | 6 |
|  | Magnesite (n=1) | 42.33 | 0.10 | 0.02 | 42.46 | 0.996 | 0.002 | 0 | 1 |
| S7126 | Pyrope (n=9) | 31.40 (0.43) | 20.11 (0.87) | 47.81 (0.61) | 99.32 (0.44) | 3.156 (0.046) | 1.598 (0.069) | 3.223 (0.033) | 12 |
|  | Stishovite (n=5) | 0 | 1.65 (0.36) | 98.48 (0.85) | 100.13 (0.69) | 0 | 0.019 (0.004) | 0.985 (0.003) | 2 |
|  | Bridgmanite (n=8) | 39.52 (0.49) | 3.26 (0.55) | 58.03 (0.92) | 100.82 (0.95) | 0.978 (0.013) | 0.064 (0.011) | 0.936 (0.007) | 3 |
|  | Melt (n=5) | 39.28 (0.43) | 3.61 (0.14) | 33.29 (0.66) | 76.18 (0.85) | 0.89 | 0.065 | 0.506 | 2 |

n represents the analyzed points for each sample.

One standard deviation of analyses is given in parenthesis.

**SI References**

1. Lu R, Keppler H. Water solubility in pyrope to 100 kbar. *Contrib Mineral Petrol* 1997; **129**: 35-42.

2. Withers AC, Wood BJ, Carroll MR. The OH content of pyrope at high pressure. *Chem Geol* 1998; **147**: 161-171.

3. Katayama I, Hirose K, Yurimoto H *et al.* Water solubility in majoritic garnet in subducting oceanic crust. *Geophys Res Lett* 2003; **30**: 2155.

4. Mookherjee M, Karato S-i. Solubility of water in pyrope-rich garnet at high pressures and temperature. *Geophys Res Lett* 2010; **37**: L03310.

5. Huang S. Influence of Chemical Composition and Water on the Bulk Modulus of Pyrope. *Doctoral Thesis*. Florida International University. 2014.

6. Thomas S-M, Wilson K, Koch-Müller M *et al.* Quantification of water in majoritic garnet. *Am Mineral* 2015; **100**: 1084-1092.

7. Panero WR, Thomas C, Myhill R *et al.* Dehydration Melting Below the Undersaturated Transition Zone. *Geochem Geophys Geosyst* 2020; **21**: e2019GC008712.

8. Liu Z, Fei H, Chen L *et al.* Bridgmanite is nearly dry at the top of the lower mantle. *Earth Planet Sci Lett* 2021; **570**: 117088.

9. Liu D, Purevjav N, Fei H *et al.* Temperature and compositional dependences of H_2_O solubility in majorite. *Am Mineral* 2024; **109**: 1646-1652.
